# Factors associated with the formation of SARS-CoV-2 case-clusters in Danish schools: a nationwide register-based observational study

**DOI:** 10.1017/S0950268823001188

**Published:** 2023-07-19

**Authors:** Tjede Funk, Laura Espenhain, Frederik Trier Møller, Steen Ethelberg

**Affiliations:** 1 Department of Infectious Disease Epidemiology and Prevention, Statens Serum Institut, Copenhagen, Denmark; 2 ECDC Fellowship Programme, Field Epidemiology Path (EPIET), European Centre for Disease Prevention and Control (ECDC), Stockholm, Sweden; 3 Department of Public Health, Global Health Section, University of Copenhagen, Copenhagen, Denmark

**Keywords:** SARS-CoV-2, cases, outbreaks, schools, Denmark

## Abstract

A register-based retrospective observational study was conducted to describe SARS-CoV-2 cases and case-clusters in schoolchildren of Danish primary and lower secondary schools and identify which factors were associated with the occurrence of case-clusters in schools. The study period was the autumn school semester 2021. Clusters were defined as three or more cases in a school-class level within 14 days. Descriptive analysis was carried out and multivariable logistic regression analysis was performed to determine which factors were associated with case introductions (i.e., primary case) being linked to a cluster. More cases and clusters were identified in lower than in higher class levels. Out of 21,497 cases introduced into a school, 41.6% started a cluster. A higher assumed immunity level in a class level was significantly reducing the odds of a case introduction being linked to a cluster (e.g., assumed immunity of ≥80% vs <20%: OR: 0.28; 95%CI: 0.17–0.44). A previous infection (in the primary case) had a protective effect (OR: 0.58; 95%CI: 0.33–0.99). This study suggests that most cases appearing in schools did not induce clusters, but that once cluster occur sizes can be large. It further indicates that vaccination of children markedly reduces the risk of secondary infections.

## Introduction

The transmission dynamics of severe acute respiratory syndrome coronavirus 2 (SARS-CoV-2) in schools have received much attention during the coronavirus-19 disease (COVID-19) pandemic. Many countries initially closed schools in response to the pandemic [1, 2], assuming that school settings might amplify community transmission. Studies from different countries have indicated low levels of transmission within school settings in the presence of certain protective measures [3–7]. This may indicate that schools in general were unlikely primary drivers of transmission in the population. Nevertheless, outbreaks in school settings have been described [8–10] and links between community incidence and risk of outbreaks in schools have been reported previously [11]. Gaining an overview of SARS-CoV-2 circulation in schools has been difficult as this has not been directly under surveillance in most countries.

To try to cast more light on this subject, we here made use of Danish register-based surveillance data. Administrative registers together with the mass testing undertaken in Denmark allow for a comprehensive mapping of SARS-CoV-2 infection in schoolchildren. During the pandemic, the Danish COVID-19 surveillance system was based on automatically captured register data. In addition, the Danish response to the COVID-19 pandemic included mass testing [12]. Denmark massively scaled up its testing capacity and ensured that every citizen had the right to be tested free of charge and independent of medical referral [13, 14]. Also in schools, testing capacities were increased and voluntary screening was implemented. During the study period, autumn school semester 2021, screening test recommendations were a main control measure implemented in school settings. When classes began in August 2021, screening was recommended for unvaccinated children from age 12 years (equivalent to approximately class level 6) and for unvaccinated staff to test twice a week (after October only once a week). On 6 September 2021, children aged 9 to 11 years (equivalent to class levels 3 to 5) were recommended to test once a week. At the end of November 2021, the recommendation was adapted to also include younger children and vaccinated children and staff [15, 16]. Based on the capacity, these screening tests (antigen tests) were conducted either directly at school premises or in one of the many available test centres (either antigen or PCR test) [16]. Further testing was recommended for persons who had contact with a confirmed case, which included inschool contact. A general infection control measure to stay at home when feeling sick or having symptoms, or after a confirmed positive test, was also implemented. Schools were kept open until 15 December 2021, after which distance schooling was implemented (for the last three days prior to the Christmas holidays) [17]. In addition, municipalities could close individual schools in consultation with the Danish Patient Safety Authority, upon finding an unmanageable SARS-CoV-2 transmission level.

To gain a national picture on and to create awareness of the spread of SARS-CoV-2 in schools, we aimed to describe SARS-CoV-2 cases and clusters in Danish primary and lower secondary schools. In addition, we sought to identify which factors were associated with a primary case being followed by a subsequent case-cluster in a class level of a school.

## Methods

### Study design and study period

This was a register-based retrospective observational study on all children in Denmark enrolled in the mandatory class levels 0 to 9 (i.e., 6 to 15 years of age) in all primary and lower secondary schools, as per Danish educational system [18]. The study period was from 9 August (first day of the autumn semester) to 19 December 2021 (last week of the school semester).

### Data sources

Data were obtained from the Danish SARS-CoV-2 surveillance which was based on linkage of different registries [19]. A list of all children attending primary and lower secondary schools in Denmark as well as their school and class level information was obtained from the Ministry of Children and Education as part of the national SARS-CoV-2 surveillance in Danish schools. Using Danish national personal identification numbers, this information was linked with other registries. We extracted all information on PCR and antigen tests from the Danish Microbiology Database, which holds information on all PCR and antigen tests conducted in Denmark (excluding self-testing, which however was not commonly applied during the study period) [20]. In addition, information on vaccination status of each schoolchild, including vaccination dates, was extracted from the Danish Vaccination Registry [21]. Finally, municipality codes of the schools made it possible to link data to incidence rates in the respective municipality.

### Definitions used

SARS-CoV-2 case: We defined a *case* as any schoolchild testing positive for SARS-CoV-2 by PCR during the study period. A person with a PCR-positive SARS-CoV-2 infection who already tested PCR-positive for SARS-CoV-2 at least 60 days prior to the current infection was considered to have a reinfection. All cases registered during the study period were included.

SARS-CoV-2 case-cluster: We defined a SARS-CoV-2 case-cluster (hereafter cluster) as ≥3 cases within 14 days in the same school and class level and spread over a period of >0 days (last sampling date – first sampling date). A cluster was considered to have ended if no new case in the school-class level had been registered for 14 days following the last case in the cluster (extending to after the end of the study period).

SARS-CoV-2 case introduction: We defined a *case introduction* as the first (or only) case registered in a class level after a period of >14 days with no SARS-CoV-2 cases registered in the same class level. That is, we considered that case to have introduced SARS-CoV-2 into the school (hereafter termed case introduction). If multiple case introductions in the same school and class level shared the same sample date and no secondary cases occurred, these were considered separate case introductions.

COVID-19 vaccination: Schoolchildren were considered vaccinated >14 days after having received the first vaccination dose.

Class-level immunity: The assumed level of immunity in each school-class level (hereafter referred to as assumed immunity level) was calculated for each day during the study period as the proportion of schoolchildren within the class level having had a positive PCR SARS-CoV-2 test in the past (also including infections prior to the study period) or having been vaccinated.

### Statistical analysis

Testing rates in school-class levels were calculated based on the number of PCR and antigen tests performed per week per 1,000 children in the respective class level. A maximum of one test per day per child was included. Weekly incidence rates in each municipality (98 in Denmark) were calculated based on national surveillance data and population sizes obtained from Statistics Denmark. Descriptive analyses were performed for the number of cases and clusters in schoolchildren. Cluster size (i.e., number of cases in the cluster), cluster length (i.e., days between first and last cases of the cluster) and attack rates (i.e. number of cases in the cluster divided by the total number of children in that school-class level) were calculated for clusters that were considered to have ended by the end of the study period.

We used multivariable logistic regression analysis to assess which factors were associated with cases starting clusters in schools. The analysis was restricted to case introductions with a unique sampling date, meaning that no other case was reported on that same day in the same school-class level. We restricted the analysis to class levels 6 to 9 since the vaccination roll-out for these children started prior to the study. The final model was adjusted for the following potential confounders: class level size, testing rate, weekly incidence in the municipality, reporting month, and province of the school. We tested for major interactions and conducted sensitivity analyses using a cluster definition of either ≥2 or ≥ 5 (instead of ≥3) cases within 14 days to assess the definition used and the robustness of the findings.

All analyses were conducted in R Statistical Software Version 4.2.1 [22].

## Results

There were 1,699 primary and lower secondary schools in Denmark in August 2021 with a total of 620,171 schoolchildren in class levels 0 to 9. During the study period, 75,225 SARS-CoV-2 infections were registered in 75,168 children (12.1% of all schoolchildren) (Table 1). A total of 5.7% (n = 35,792) of schoolchildren had already been infected prior to the study period. During the study period, at least one SARS-CoV-2 case was registered in 96.2% of schools (n = 1,634), while 76.5% of schools (n = 1,300) had at least one cluster. By 19 December 2021, 2.5% of children in class levels 0 to 4 and 63.1% of children in class levels 5 to 9 were vaccinated. Almost all schoolchildren (94.4%) were tested at least once during the study period.

**Table 1.** Characteristics of schoolchildren, cases, and case introductions

Variable	Schoolchildren	Cases	Case introductions
	n (%)	n (%)	n (%)
**Total**	620,171	75,225^±^	21,497^¶^
Vaccination status*			
Not vaccinated	408,702 (65.9)	70,009 (93.1)	19,551 (90.9)
Vaccinated	211,469 (34.1)	5,216 (6.9)	1,946 (9.1)
**Part of clusters**			
Yes	55,888 (9.0)	55,912 (74.3)	8,949 (41.6)
No	564,283 (91.0)	19,313 (25.7)	12,548 (58.4)
**Class level**			
0–5	362,284 (58.4)	57,607 (76.6)	14,112 (65.6)
6–9	257,887 (41.6)	17,618 (23.4)	7,385 (34.4)
**Class level size**			
0–25	102,571 (16.5)	10,986 (14.6)	5,064 (23.6)
26–50	167,258 (27)	19,214 (25.5)	6,367 (29.6)
51–75	194,218 (31.3)	24,731 (32.9)	6,173 (28.7)
76–100	104,424 (16.8)	14,268 (19)	2,839 (13.2)
>100	51,700 (8.3)	6,026 (8)	1,054 (4.9)
**Province**			
Bornholm	3,685 (0.6)	422 (0.6)	145 (0.7)
Copenhagen City	70,905 (11.4)	14,077 (18.7)	3,108 (14.5)
Copenhagen surroundings	64,665 (10.4)	11,798 (15.7)	2,580 (12)
East Jutland	97,182 (15.7)	8,530 (11.3)	2,719 (12.6)
East Zealand	30,452 (4.9)	4,099 (5.4)	1,186 (5.5)
Funen	51,568 (8.3)	4,784 (6.4)	1,589 (7.4)
North Jutland	61,088 (9.9)	5,071 (6.7)	1,808 (8.4)
North Zealand	54,121 (8.7)	7,621 (10.1)	1,955 (9.1)
South Jutland	79,464 (12.8)	7,549 (10)	2,571 (12)
West and South Zealand	59,093 (9.5)	6,873 (9.1)	2,302 (10.7)
West Jutland	47,948 (7.7)	4,401 (5.9)	1,534 (7.1)

*Note:* ±Includes 57 schoolchildren that were infected twice ¶ Includes 6 schoolchildren that were infected twice *For schoolchildren, the vaccination status was calculated at the end of the study period, and for cases and case introductions, it is at the time of infection during the study period.

### Cases and clusters in schools

**Figure 1.**
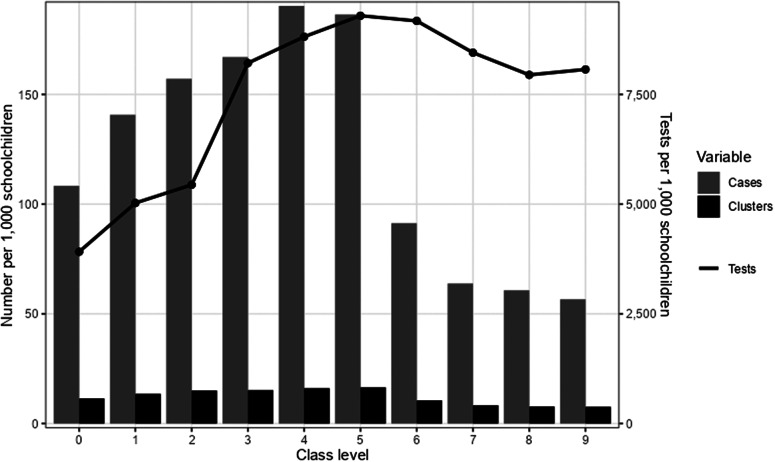
Number of cases, clusters and tests per 1,000 schoolchildren per class level.

A total of 7,518 clusters in schools were identified, encompassing 55,912 cases (74.4% of all cases) (Tables 1 and 2). Of all clusters, 54.4% (n = 4,090) had ended by 19 December 2021 (Table 2). Clusters ranged in size between 3 and 65 cases (median: 5 cases; IQR: 3–9 cases), and the length varied between 1 and 61 days (median: 10 days; IQR: 6–16 days). Overall, more clusters were reported in class levels 0 to 5 than in 6 to 9 (14.7 vs. 8.5 per 1,000 schoolchildren, respectively) and the median size of clusters was larger in lower class levels (Table 2). Attack rates of clusters ranged from affecting 1.3% to 100% (median 13.3%; IQR: 7.3–23.8%) of the class level. Looking at all class levels separately, the number of cases per 1,000 schoolchildren and clusters per 1,000 schoolchildren increased from class levels 0 to 4, while testing per 1,000 schoolchildren was higher in higher class levels than in lower class levels (Figure 1). The number of cases, clusters and tests performed increased towards the end of the study period (Supplementary Materials 1–3).

**Table 2.** Characteristics of clusters by class level groups

Variable	Total	Class level 0–5	Class level 6–9
**Total number of clusters**	7,518	5,314	2,204
**Clusters per 1,000 schoolchildren**	8.6	14.7	8.5
**Cluster status**			
Over	4,090 (54.4%)	2,998 (56.4%)	1,092 (49.5%)
Ongoing	3,428 (45.6%)	2,316 (43.6%)	1,112 (50.5%)
**Size (cases)**			
Median (IQR)	5 (3–9)	6 (4–10)	4 (3–7)
Range (min, max)	3, 65	3, 65	3, 30
**Length (days)**			
Median (IQR)	10 (6–16)	10 (6–16)	9 (5–14)
Range (min, max)	1, 61	1, 60	1, 61
**Attack rate (%)**			
Median (IQR)	13.3 (7.3–23.8)	15.55 (8.6–27.5)	8.5 (5.4–15.2)
Range (min, max)	1.3, 100	2.1, 100	1.3, 75

### Case introductions and their link to clusters

There were 21,497 case introductions identified. Two-thirds (65.6%, n = 14,112) of these were in class levels 0 to 5 (Table 1). Eighty-five per cent (n = 18,476) of the case introductions had a unique sample date, of which 6,526 occurred in class levels 6 to 9 and were included in the analysis of determinants for clusters to occur. Of all case introductions, 41.6% were linked to clusters (34% of case introductions with unique sample dates were linked to clusters).

The results of the logistic regression analysis are presented in Table 3. The adjusted odds of starting a cluster were lower for vaccinated than for non-vaccinated case introductions (OR: 0.84, 95%CI: 0.72–0.97) (Table 3). However, this finding was not consistent when using other cluster definitions (OR: 0.9; 95%CI: 0.78–1.03, for the cluster definition of ≥2 cases; OR: 1.04; 95%CI: 0.85–1.28, for the cluster definition of ≥5 cases). An increased assumed immunity level in the class level reduced the odds of starting a cluster (e.g., assumed immunity of ≥80% compared with <20%: OR: 0.28; 95%CI: 0.17–0.44) (Table 3). The same effect was seen in the model using the cluster definition of ≥2 cases (≥80% compared with <20%: OR: 0.3; 95%CI: 0.2–0.45) and ≥ 5 cases (≥80% compared with <20%: OR: 0.11; 95%CI: 0.06–0.2). A case introduction who had a previous SARS-CoV-2 infection had marginally reduced odds of starting a cluster (OR: 0.58, 95%CI: 0.33–0.99). Despite the odds also being reduced in the sensitivity analyses, they did not reach significance (OR: 0.91; 95%CI: 0.57–1.47, for the cluster definition of ≥2 cases; OR: 0.76; 95%CI: 0.35–1.5, for the cluster definition of ≥5 cases). An increase in class level size, incidence in the municipality, testing rate, and a reporting month later in the year all increased the odds of a case introduction starting a cluster (Table 3).

**Table 3.** Adjusted odds ratios of factors associated with case introductions in school-class levels 6 to 9 (n = 6,526) being linked to a cluster, Denmark, 2021

Variable	n	Adjusted odds ratio (95% CI)	*p*-value
**Vaccination status of case**			
Not vaccinated	4,839	Ref	Ref
Vaccinated	1,687	0.84 (0.72–0.97)	0.021
**Previous infection**			
No	6,437	Ref	Ref
Yes	89	0.58 (0.33–0.99)	0.051
**Assumed immunity level (%)**			
0–19	169	Ref	Ref
20–39	706	1.08 (0.71–1.67)	0.714
40–59	1,361	0.63 (0.41–0.98)	0.037
60–79	2,503	0.37 (0.24–0.58)	<0.001
≥80	1,787	0.28 (0.17–0.44)	<0.001
**Class level size**			
0–25	1,159	Ref	Ref
26–50	1,687	2.95 (2.39–3.66)	<0.001
51–75	2,050	4.52 (3.66–5.61)	<0.001
76–100	1,121	7.01 (5.54–8.91)	<0.001
>100	509	12.53 (9.48–16.61)	<0.001
**Incidence in municipality (by week per 1,000 population)**			
0–4	3,617	Ref	Ref
5–9	2,001	1.32 (1.09–1.6)	0.005
10–14	549	2.55 (1.9–3.44)	<0.001
15+	359	6 (4.19–8.62)	<0.001
**Testing rate per 1,000**			
<500	2,059	Ref	Ref
500–999	2,505	1.41 (1.21–1.65)	<0.001
1,000–1,449	1,297	1.82 (1.51–2.19)	<0.001
≥1500	665	3.14 (2.5–3.96)	<0.001
**Reporting month**			
August	946	Ref	Ref
September	423	1.02 (0.72–1.44)	0.91
October	976	1.77 (1.34–2.35)	<0.001
November	2,129	2.88 (2.21–3.77)	<0.001
December	2,052	5.16 (3.69–7.25)	<0.001

*Note*: The table presents adjusted odds ratios. The model also adjusted for 11 provinces (data not shown here).

Abbreviations: Ref: Reference group.

## Discussion

This study was a register-based retrospective cohort study including all schoolchildren in Denmark, a population group for which official routine testing recommendations were introduced during the study period (the autumn semester of 2021). This study showed that cases were identified in nearly all schools throughout the country and that, using our cluster definition, clusters developed in 3 out of 4 schools. More cases and clusters per schoolchild were found in class levels 0 to 5 (i.e., younger children), where vaccination roll-out did not begin until late November 2021. Clusters in higher class levels were overall smaller and had a lower attack rate. Forty-two per cent of all case introductions were linked to a subsequent cluster, while overall 74% of all cases were linked to clusters. Multivariable logistic regression analysis showed that the odds of a case introduction in class levels 6 to 9 starting a cluster was significantly reduced if the case introduction went to a school-class level with a high assumed immunity level. In addition, the vaccination status of the case introduction and a previous infection reduced the odds, though this finding was sensitive to which cluster definition we used.

More cases and clusters per 1,000 children were reported in lower class levels than in higher class levels, while testing rates were higher in higher class levels. This does not seem surprising considering that young children were susceptible throughout the majority of the study period as vaccination roll-out for children 5 to 11 years did not begin until the end of November [23]. A similar trend was seen in the incidence rates in the general Danish population, where the age group 6 to 11 years had the highest incidence among the age groups during the study period [24, 25]. National overviews on the occurrence of cases and clusters in schools, including testing rates and vaccination coverage, are, to our knowledge, still scarce in the literature. However, our findings are consistent with data from Norway where more clusters occurred in primary schools than in secondary schools [25, 26].

Incidence rates of the municipality were associated with clusters in schools. This makes sense as increased SARS-CoV-2 circulation in the community will increase the likelihood that a case is introduced into a school and clusters occur. An association between community incidence and clusters in educational settings has been found in other studies [11, 25]. Unsurprisingly, variables that were first and foremost included in the model to remove their confounding effect such as a larger class level size and higher testing rates also substantially increased the odds of a case introduction being linked to a cluster.

More than half of the case introductions (58.4%) in our study were not linked to a cluster, a finding that is supported by other studies too [27–29]. This suggests that not only can sporadic cases occur in a class level without onward transmission but also that it can occur in a setting with few protective measures in place as in Denmark. Our study additionally found that in contrast to case introductions, 74% of all cases during the study period were part of clusters.

Our findings further strongly suggest that vaccination prevented clusters. There was a clear correlation between an increased, assumed immunity levels in a school-class level resulting in lower odds of an infected child starting a cluster. Much research has been dedicated to the COVID-19 vaccine effectiveness during different stages of the pandemic. In the context of schools and schoolchildren, vaccines against COVID-19 were found to reduce the risk of infections in modelling [30, 31] and observational studies [32–34]. It has also been suggested that previous infections in children and adolescents may enhance the protective effect against infection received through vaccination [32]. There are fewer studies assessing the effect of vaccination of the primary or index case on secondary transmission in schools in particular; however, in the context of households, the effect of a vaccinated primary case on secondary transmission seems to vary in different studies between no effect and a protective effect [33, 35–37]. Likewise, in our study, the role of vaccination of the case introduction itself on the risk of secondary transmission remains unclear as shown by the inconsistency between the findings in our main analysis compared with the sensitivity analyses. How a potential varying test activity or symptomatology in vaccinated and unvaccinated may have affected the results is unknown. The protective effect of a previous infection in case introductions on starting a cluster was in this study marginally significant, but not significant in the sensitivity analyses. This could be explained by low number of observations.

## Strengths and limitations

Denmark has high-quality register data covering the entire population, and in combination with extensive testing efforts in the Danish population and testing recommendations in schools specifically, this provided a good opportunity for studying SARS-CoV-2 cases and clusters in schools. However, this also entails that the cluster definition used in this study is based on register data only and on the assumption that cases occurring closely in time and space are linked to the school. Similar approaches to defining outbreaks based on case counts and registers have been used in, for instance, Norway [26] and England [11]. In order to increase the specificity of our cluster definition, we defined clusters by class level, as opposed to the whole school as previously used by others [11, 26]. Nevertheless, we defined clusters as three or more cases within 14 days without including information from whole genome sequencing and transmission links outside the school setting (e.g., household links). Including these variables could not only have affected the number and size of clusters but also impacted the school transmission observed in this study in general.

Generally, younger schoolchildren were not included in the recommendation on weekly screening in Denmark and were therefore tested less frequently than older schoolchildren. Although testing as part of contact-tracing efforts was recommended for everyone as opposed to quarantine, younger schoolchildren still got tested less frequently than older schoolchildren. The difference in testing activity makes comparisons across class levels more difficult and could have contributed to an underestimation of the number of cases in the lowest class levels.

This study defined a case as a PCR-confirmed SARS-CoV-2 test only, despite antigen testing taking place too. At the time of the study, schoolchildren were recommended to confirm a positive antigen test result by PCR in order to get out of quarantine, should the antigen test result be false positive. We therefore considered our definition as the more reliable choice. While, the choice of this method might result in individual cases being missed, false-positives test results are thereby also excluded.

In addition, this study was based on the assumption that the first person who tested positive is the case introduction in the class level (i.e., introduced the disease into the class level and led to additional cases) and that clusters involving only cases with the same sample date are separate introductions and not linked. This might however not always be true, as children may have experienced asymptomatic infections and not necessarily got tested before prompted by the identification of another case in the class. Consequently, asymptomatic cases might have been missed and some clusters misclassified. Due to the weekly testing recommendations in place, this may however have been less of an issue in Denmark than in other settings. Furthermore, this study was conducted in the period when the SARS-CoV-2 Delta variant was dominant, which, in contrast to the Omicron variant, was more often causing symptomatic infections [38].

This study only focused on schoolchildren and not school staff as we were not able to link teachers or other staff to specific class levels within a school. For this reason, we were not able to assess potential differences in onward transmission from child versus adult case introductions.

## Conclusion

Our study showed that between August and December 2021, when the Delta variant was dominant, nearly all schools had seen SARS-CoV-2 cases while 77% had seen clusters of cases. Overall, more cases and clusters had been seen in the lower class levels, where children were mainly unvaccinated, than in higher class levels. More than half of the case introductions identified in this study were not followed by a subsequent cluster of cases, while 75% of all cases were part of clusters. The assumed immunity level in the class level (i.e., vaccination coverage and previous infection) had a significant protective effect on cluster building in school settings, indicating that vaccinations effectively prevented cluster formation.

## Data Availability

De-identified data are available for access to members of the scientific community for noncommercial use. More information, including on how to apply, is available through *Forskerservice* at The Danish Health Data Authority. Applications will be reviewed on the basis of relevance and scientific merit.

## Supplementary material

For supplementary material accompanying this paper visit http://doi.org/10.1017/S0950268823001188. click here to view supplementary material
