# Supplementary material for: Factors associated with the formation of SARS-CoV-2 case-clusters in Danish schools: a nationwide register-based observational study
Source: Epidemiol Infect. 2023 Jul 19;151:e168. doi: 10.1017/S0950268823001188 (PMC10600729; doi:10.1017/S0950268823001188)

# *Epidemiology and Infection*

**Factors associated with SARS-CoV-2 formation in Danish schools: a nationwide register-based cohort study**

Tjede Funk, Laura Espenhain, Frederik Trier-Møller, Steen Ethelberg

# Supplementary material

Supplementary Figure S1. Number of cases per 1000 school children per reporting week in lower and higher class levels

Supplementary Figure S2. Number of clusters per 1000 school children per reporting week in lower and higher class levels

Supplementary Figure S3. Number of tests (PCR and antigen combined) per 1000 school children per reporting week in lower and higher class levels

Supplementary Figure S4. Vaccination coverage of school children over study period

Supplementary Figure S1. Number of cases per 1000 school children per reporting week in lower and higher class levels

**
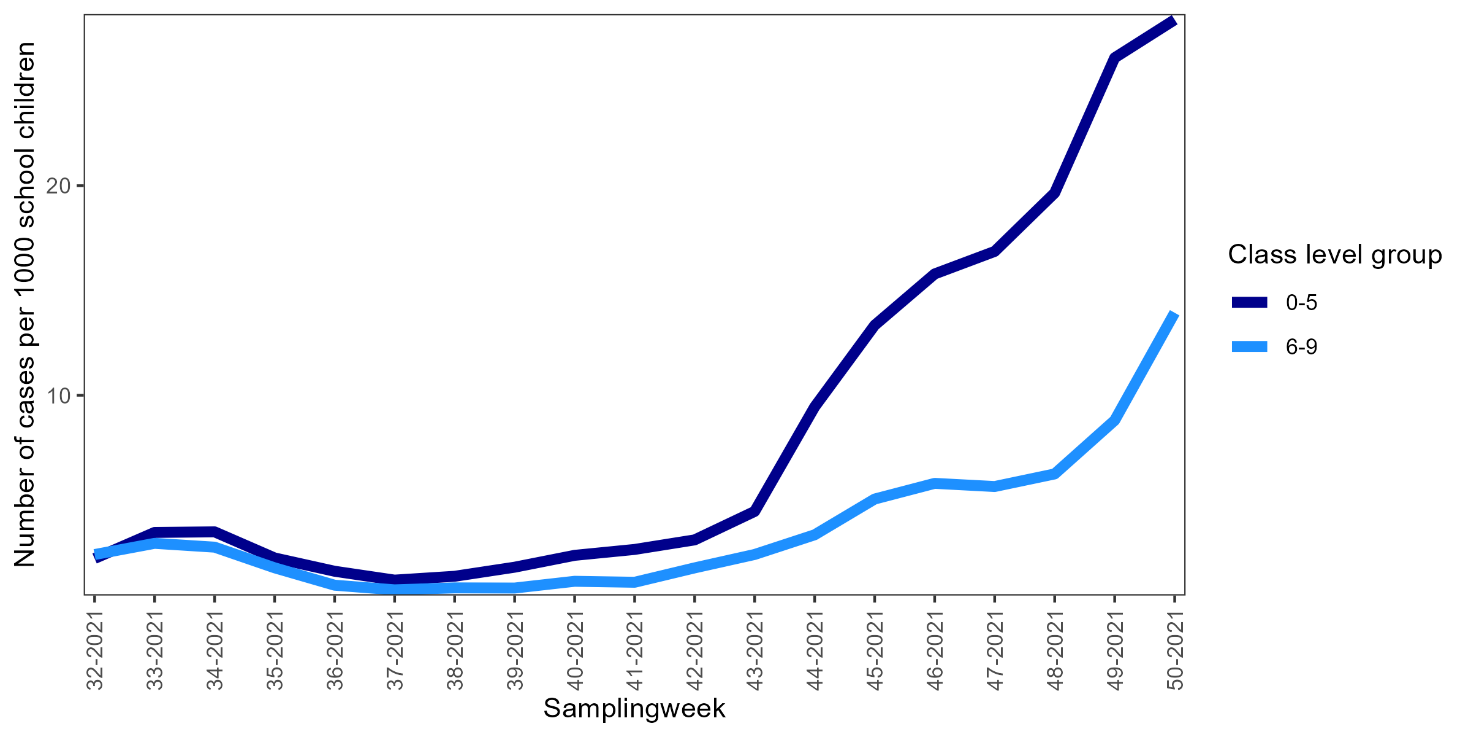
**

*Note that in week 42 was the week of the school autumn holidays*

Supplementary Figure S2. Number of clusters per 1000 school children per reporting week in lower and higher class levels


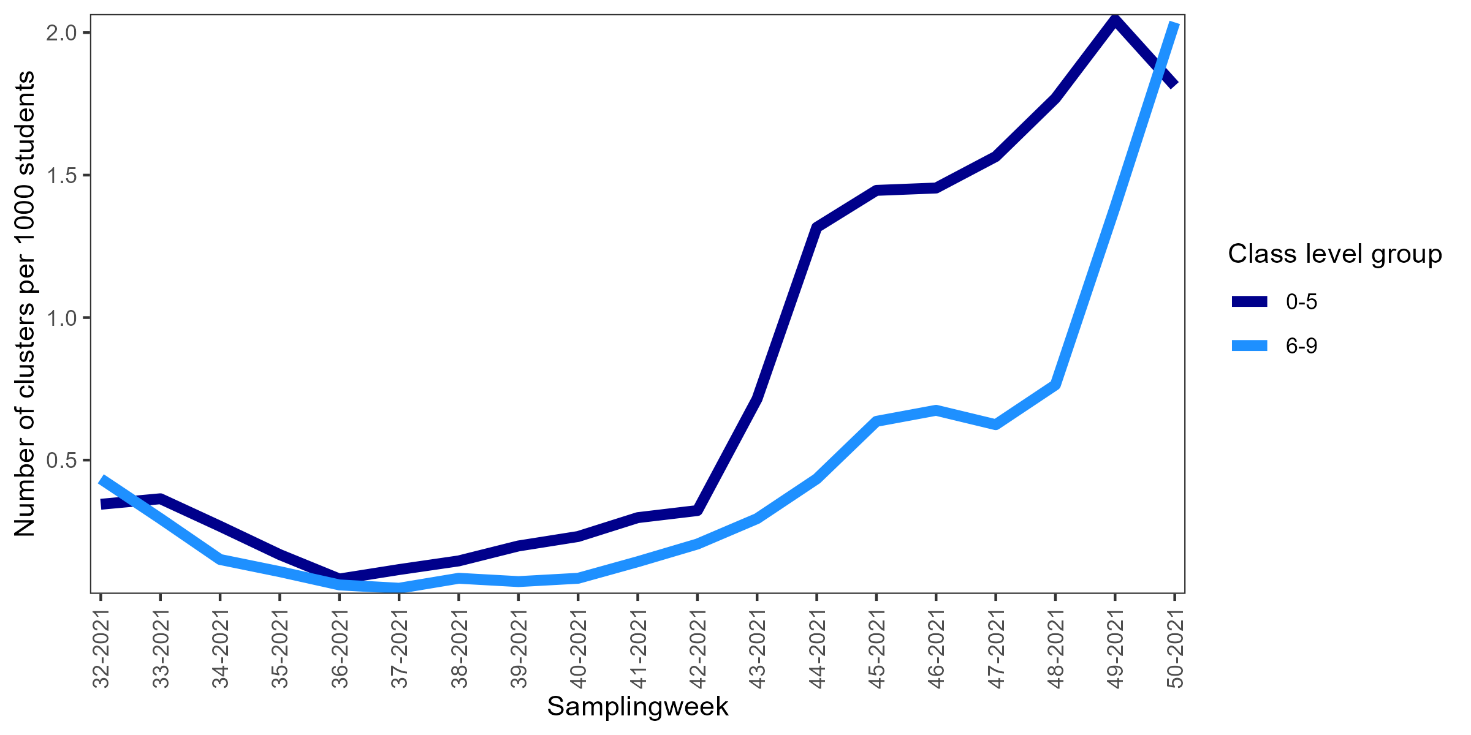


*Note that in week 42 was the week of the school autumn holidays*

Supplementary Figure S3. Number of tests (PCR and antigen combined) per 1000 school children per reporting week in lower and higher class levels


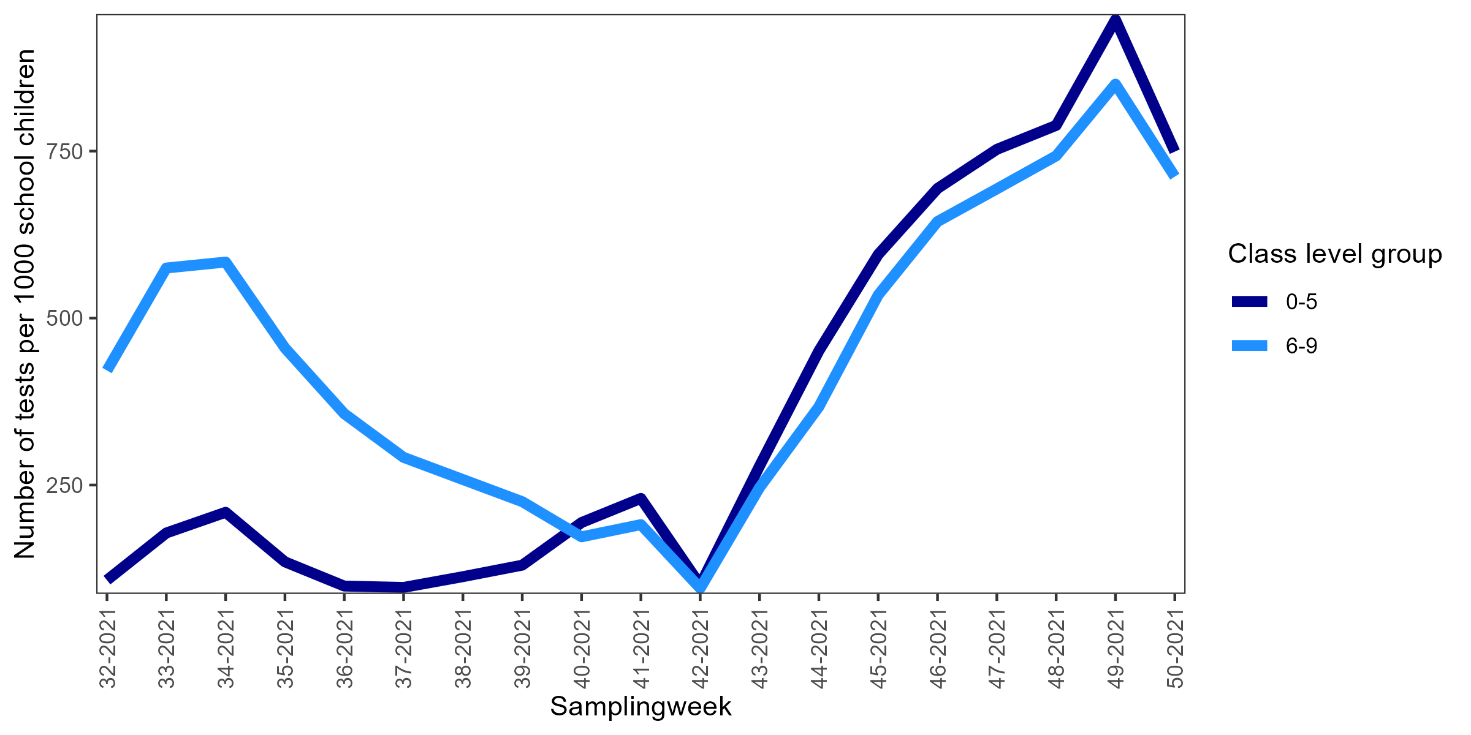


*Note that in week 42 was the week of the school autumn holidays*

Supplementary Figure S4. Vaccination coverage of school children over study period


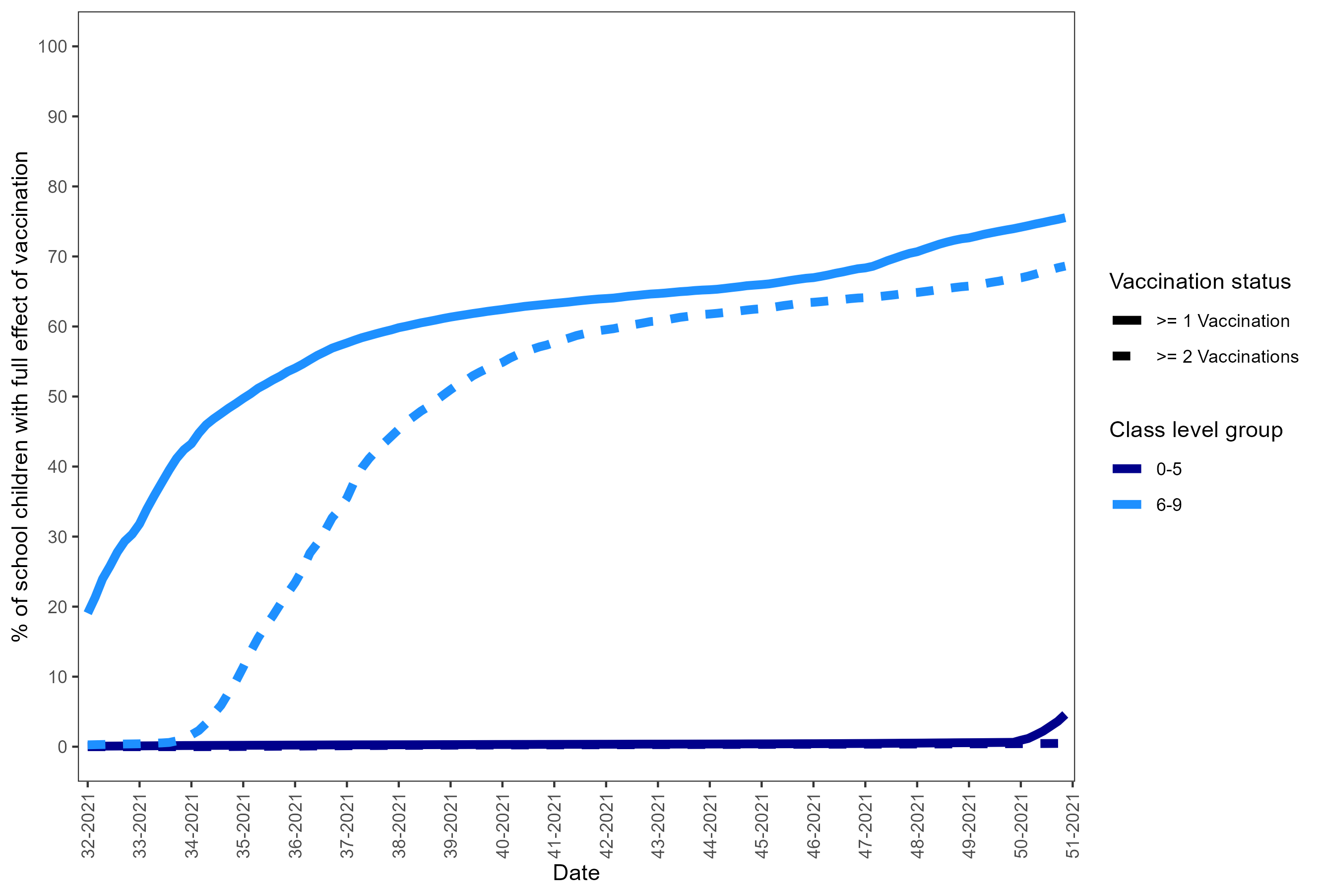

Supplement: Supplementary file 1 [file S0950268823001188sup001.docx]
